# Predicting herd immunity achievement: a time-series analysis of vaccination and fatality rates using 1,075 days of COVID-19 data

**DOI:** 10.3389/fpubh.2024.1403163

**Published:** 2024-09-20

**Authors:** Benny Yiu Chung Hon, Jeffrey Chan, Kei Shing Ng, Simon Ching Lam

**Affiliations:** ^1^Department of Mathematics, Faculty of Science, The Chinese University of Hong Kong, Sha Tin, Hong Kong SAR, China; ^2^Department of Psychology, School of Humanities and Social Sciences, University of Science and Technology of China, Hefei, China; ^3^King George V School, Ho Man Tin, Hong Kong SAR, China; ^4^Department of Diagnostic Radiology, Li Ka Shing Faculty of Medicine, The University of Hong Kong, Pok Fu Lam, Hong Kong SAR, China; ^5^School of Nursing, Tung Wah College, Ho Man Tin, Hong Kong SAR, China

**Keywords:** COVID-19, vaccine hesitancy, herd immunity, time series analysis, BNT162b2, CoronaVac

## Abstract

**Introduction:**

The COVID-19 pandemic, driven by SARS-CoV-2, has made vaccination a critical strategy for global control. However, vaccine hesitancy, particularly among certain age groups, remains a significant barrier to achieving herd immunity.

**Methods:**

This study uses Poisson regression and ARIMA time-series modeling to identify factors contributing to vaccine hesitancy, understand age-specific vaccination preferences, and assess the impact of bivalent vaccines on reducing hesitancy and fatality rates. It also predicts the time required to achieve herd immunity by analyzing factors such as vaccine dosing intervals, age-specific preferences, and changes in fatality rates.

**Results:**

The study finds that individuals recovering from COVID-19 often delay vaccination due to perceived immunity. There is a preference for combining BNT162b2 and CoronaVac vaccines. The BNT162b2 bivalent vaccine has significantly reduced vaccine hesitancy and is linked with lower fatality rates, particularly in those aged 80 and above. However, it tends to induce more severe side effects compared to Sinovac. Vaccine hesitancy is most prevalent among the youngest (0–11) and oldest (80+) age groups, posing a challenge to reaching 90% vaccination coverage.

**Conclusion:**

Vaccine hesitancy is a major obstacle to herd immunity. Effective strategies include creating urgency, offering incentives, and prioritizing vulnerable age groups. Despite these challenges, the government should have continued to encourage vaccinations while gradually lifting COVID-19 control measures, balancing public health safety with the return to normal life, as was observed in the transition period during the latter stages of the pandemic.

## Introduction

A coronavirus, known as SARS-CoV-2, was a new strain of the coronavirus family that used RNA as its genetic material to cause the disease COVID-19. It was transmitted primarily through airborne (1) particles and direct contact, causing flu-like symptoms: cough, loss of taste or smell, headache, and more. With this rampant virus spread, various countermeasures, including vaccinations, social distancing, mask-wearing, and quarantine measures, have been crucial in controlling the virus.

Additionally, factors within the social determinants of health, such as access to healthcare, socioeconomic status, education, and public awareness, play significant roles in the effectiveness of these measures.

Until December 31, 2022, over 660 million COVID-19 positive cases and 6.69 million deaths were recorded worldwide, of which 2.62 million COVID-19 positive cases and 11,000 deaths were recorded in Hong Kong (2). However, some people were hesitant (3) to receive the vaccine, as indicated by the low vaccination rates, which warranted an investigation. In other settings, factors contributing to COVID-19 vaccine hesitancy include concerns about vaccine safety and side effects, misinformation and conspiracy theories, lack of trust in government or healthcare systems, and cultural or religious beliefs. In this study, statistical analysis of government data will provide a suitable conclusion to address why certain age groups were vaccine-hesitant and what percentage of individuals were required to be vaccinated to yield herd immunity (4). Achieving herd immunity is crucial during the COVID-19 pandemic because it significantly reduces the spread of the virus, thereby protecting those who cannot be vaccinated, such as individuals with certain medical conditions or those ineligible for the vaccine. Herd immunity can help prevent healthcare systems from becoming overwhelmed and reduce the overall mortality and morbidity associated with the virus.

**Figure 1:**
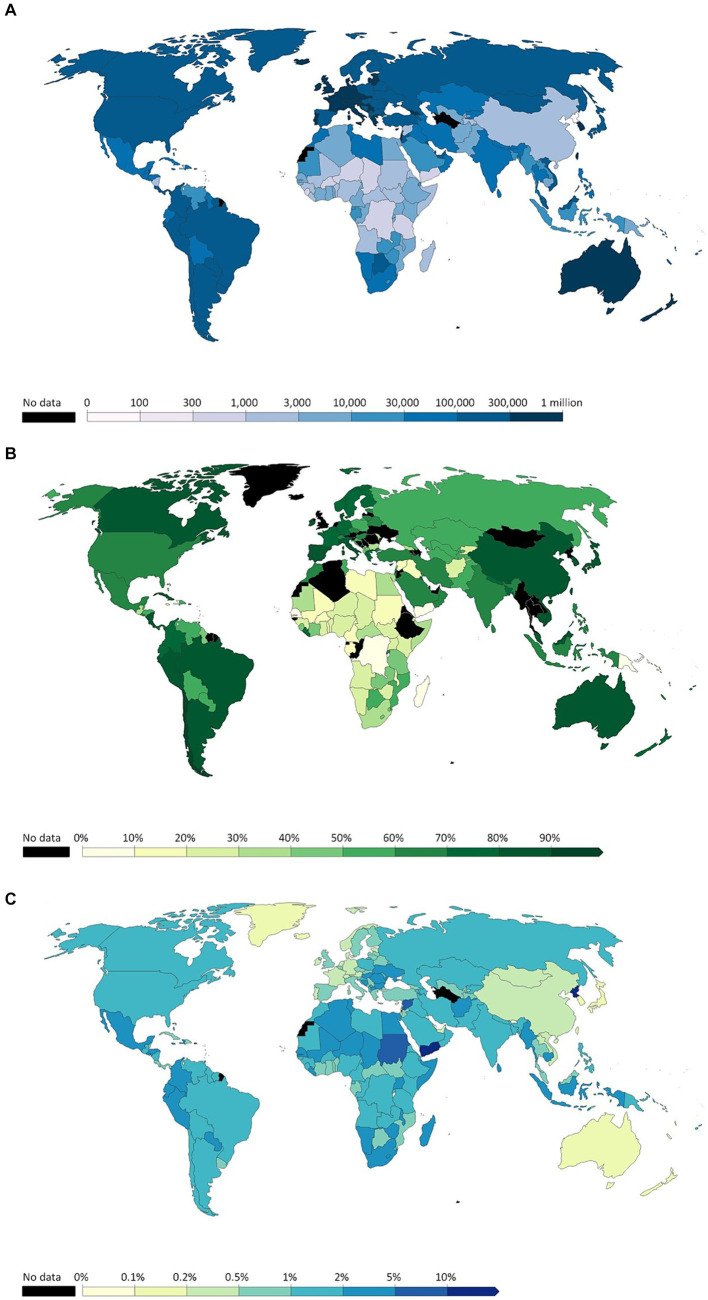
**(A)** Cumulative confirmed COVID-19 cases per million people before December 31, 2022. **(B)** Share of people who completed the initial COVID-19 vaccination protocol before December 31, 2022. **(C)** Cumulative mortality rate before December 31, 2022 (Adopted from: https://ourworldindata.org/covid-cases).

Figure 1A shows that Australia and some parts of Western Europe, including Iceland, had the greatest number of cases, represented by the darkest blue in this heatmap. They are followed by North and South America and Russia, shown in the second darkest blue. Figure 1B shows that China, Australia, Canada, most of South America, and most of Western Europe had a vaccination rate of 80% or higher. However, most of Africa, in its northern countries, had a low vaccination rate ranging from mostly being close to 0% to a few having a vaccination rate of nearly 60%. Figure 1C shows that Australia, China, Mongolia, Greenland, and Western Europe had very low mortality rates. North and South America had a low mortality rate, between 1 and 2%. However, Northern Africa had a high mortality rate, especially Yemen with a 10% or higher mortality from COVID-19. Additionally, North Korea had a COVID-19 mortality rate of 10% or higher. The heatmaps in Figure 1 indicate that a higher vaccination rate generally led to lower COVID-19 mortality rates and vice versa, due to the effectiveness of modern COVID-19 vaccines at reducing the chance of COVID-19 complications.

**Figure 2:**
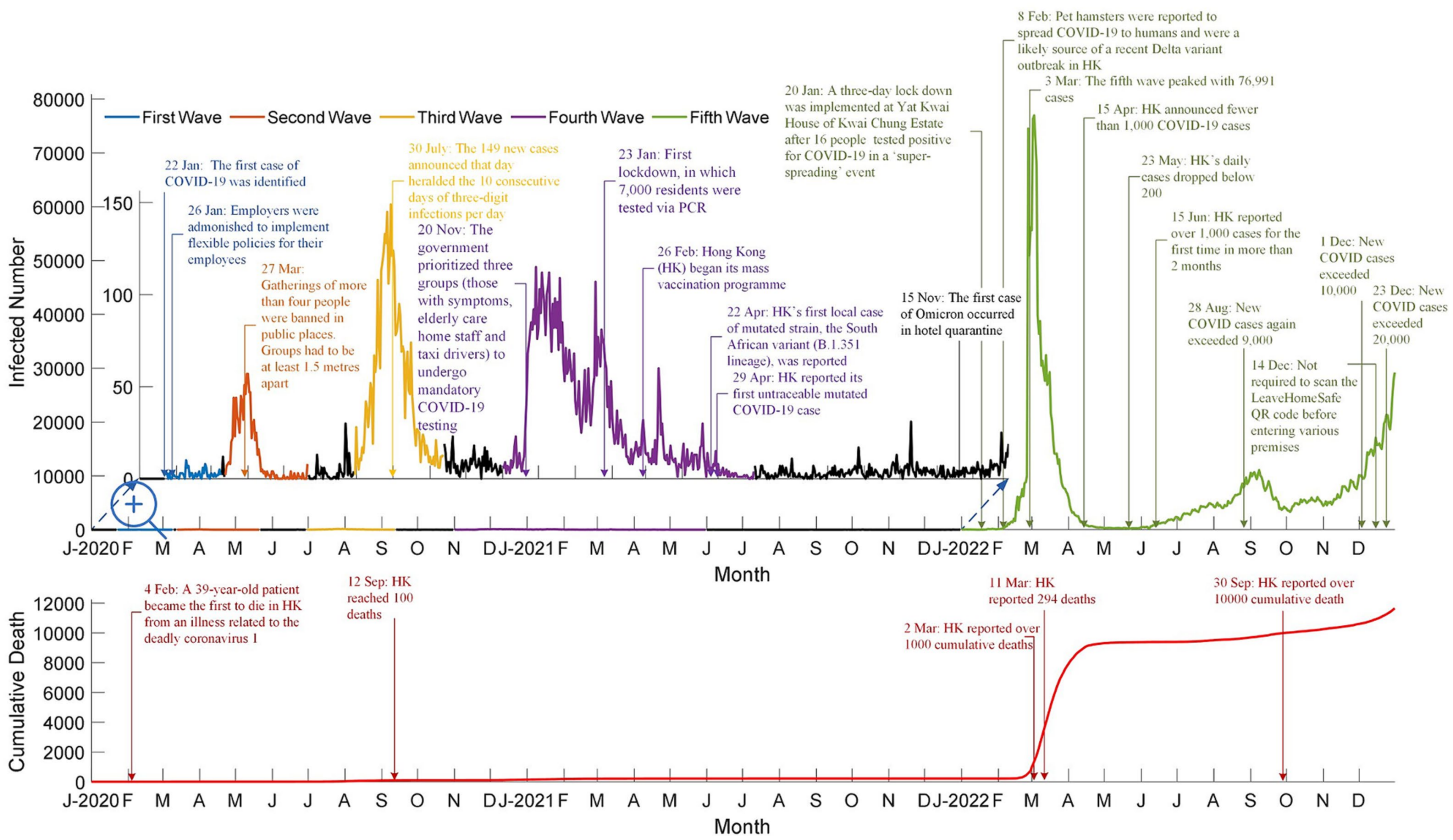
Number of daily infected cases, cumulative death cases, and highlights for the first five waves in Hong Kong (Adpoted from: https://data.gov.hk/en-data/dataset/hk-dh-chpsebcddr-novel-infectious-agent).

With this trend in mind, evidence on the effectiveness of vaccines should have been sufficient to assume that Hong Kong would have obtained a high vaccination rate, yet this is not the case. Hong Kong’s vaccination rate was low due to vaccine hesitancy among specific age groups, particularly parents making vaccination decisions for their children (5). Figure 2 and Supplementary Table S1 present detailed data on the COVID-19 pandemic in Hong Kong during its first five waves. These include the number of daily infections, cumulative fatalities, significant incidents in Hong Kong, and government actions, as well as the number of cases resulting in death and the dominant variants for each wave.

Vaccination programs were implemented during the fourth wave to reduce COVID-19’s mortality rate. These programs included free vaccinations for the public and priority provided to specific groups, such as the older adult, health workers, and chronically ill patients. Such programs and strategic vaccination priorities, along with other factors such as selective mortality, increased general awareness, and public health interventions, contributed to lowering the mortality rate from 2.5% in the third wave, to 1.79% in the fourth wave, and then finally to 0.44% in the fifth wave. The decreased mortality rate after the vaccination program substantiates how vaccines reduce the number of severe complications and COVID-19-related deaths.

This study begins with a summary of the pandemic situations, followed by a segment on data collections and statistical methods used to investigate the factors contributing to vaccine hesitancy. After that, the main statistical results are presented, followed by a discussion of the key findings related to vaccine hesitancy. The study concludes with a section offering potential suggestions to minimize vaccination hesitancy in Hong Kong.

## Methods

This study is based on a secondary data analysis of extensive datasets obtained from official databases in Hong Kong, specifically https://www.coronavirus.gov.hk/ and https://data.gov.hk/en-data/dataset/hk-dh-chpsebcddr-novel-infectious-agent. The data management process encompassed several key stages: initial data collection and retrieval, thorough data cleaning, and a comprehensive four-phase data analysis approach, each phase employing suitable statistical methods tailored to the specific nature of the data.

The statistical analysis included the use of ARIMA models to predict vaccination trends and the impact of various factors on vaccination rates. Detailed information about the specific statistical methods, tests, models, and assumptions employed in the analysis is provided in the supplementary material. Readers are encouraged to refer to the supplementary material A for a comprehensive understanding of the rigor and validity of the analysis.

### Data collections

The COVID-19 data was provided by the Hong Kong Government from the 23rd of January 2020 to the 31st of December 2022. This data included the daily infected cases and death cases from the 23rd of January 2020 to the 31st of December 2022 (1,075 days), and the vaccination number from the 26th of February 2021 to the 31st of December 2022 (678 days).

In addition to sourcing the data from the government, data cleansing (6) is performed to correct the minor errors present in the data sources.

### Data cleaning

The data cleaning process involved several steps to ensure accuracy and reliability. First, we identified and corrected inaccuracies by cross-referencing with multiple sources, including media reports and press releases from the Centre for Health Protection (CHP). We addressed formatting issues by standardizing date formats and ensuring consistent naming conventions across datasets. Duplicate entries were detected and removed by checking for repeated records with identical timestamps and case details. Incomplete data entries were either supplemented with information from reliable sources or excluded if necessary. We employed specific algorithms to merge multiple data sources, ensuring the integration process maintained data integrity and consistency.

### Data analysis methods

Statistical analyses were performed using SPSS (IBM SPSS Version 29). Data visualizations were performed using MATLAB (MATLAB R2024a; MathWorks Inc., Natick, MA, USA). Statistical significance was considered at *p*-value <0.05.

This study encompasses five distinct analyses, each focusing on a different aspect of the COVID-19 vaccination process and its effects on vaccination rates, public health outcomes, and vaccine hesitancy in Hong Kong.

#### Analysis 1

The second dose should have been received 21 days after the first dose to guarentee full protection by vaccination. To test the hypothesis that people were unwilling to take the second dose in 2021 because most of the infected cases were from imported cases, we conducted a time-series analysis to compare the vaccination rates with the daily infection rates. This analysis assessed whether there was a significant correlation between low locally reported infection cases and increased vaccine hesitancy. Therefore, vaccination hesitancy was due to the low number of infected cases reported locally, as supported by empirical data indicating that regions with fewer reported cases, like Hong Kong, experienced higher levels of vaccine complacency and hesitancy (7, 8).

#### Analysis 2

The brand of the vaccines was one of the deterministic factors for willingness to receive the vaccine. A Chi-square test for independence was conducted to examine this relationship. Groups aged 3–11 and age 80 or above had the lowest vaccination rates due to fear of side effects. This is not unexpected, as vulnerability is a crucial concept in public health interventions. Both very young children and older adults are more likely to experience severe side effects, leading to higher hesitancy in these groups (9, 10).

#### Analysis 3

The government launched *Comirnaty Original/Omicron BA.4/BA.5 bivalent* vaccines from December 1, 2022. Using Poisson regression for count (11), the impact of the number of vaccinations a week before and after the launch of the bivalent vaccines can be quantified. The introduction of new vaccines specifically targeting the Omicron variant positively influenced vaccination uptake by reducing hesitancy among individuals concerned about the variant’s spread.

#### Analysis 4

Vaccination can effectively reduce COVID-19 symptoms and especially decrease the fatality rate, lower in vaccinated than unvaccinated population. The Chi-square test for independence (12) is used to investigate any association between fatality number and vaccine type administered, providing insights into the effectiveness of different vaccines and informing strategies to improve vaccination rates and public health outcomes.

#### Analysis 5

Time-series analysis (13) using the ARIMA model is conducted to predict the time required to achieve herd immunity and to declare the pandemic becoming an endemic. All COVID-19 restrictions could have been loosened or canceled once the community achieved herd immunity. The ARIMA model is referred to mathematically or symbolically as an ARIMA (*p*, *d* and *q*) model, where *p*, *d* and *q* are nonnegative values and stand for the orders of the autoregression, differencing and moving average parts of the model, respectively. The best ARIMA model was selected using the Expert Modeler feature in IBM SPSS software, which automatically identifies the optimal model parameters based on several fit criteria, including the Bayesian Information Criterion (BIC), Akaike Information Criterion (AIC), and the stationary R-square. The software evaluates different combinations of autoregressive (*p*), differencing (*d*), and moving average (*q*) components and selects the model that best fits the data according to these statistical criteria. An important component of the Box–Jenkins approach to time arrangement presentation includes the ARIMA models. The best time series model for the current investigation is selected using the expert modeler of IBM SPSS 29 Software.

## Results

### Analysis 1

Supplementary Figure S1 presents the daily infected cases and the vaccination rate for the first five waves. It shows that 60% of people took the first two doses since the launch of the vaccination program (14). The vaccination was not mandatory until the 24th of February 2022, when the daily number of infected cases reached its maximum in early March 2022. The government launched the third dose in late 2021, and the cumulative vaccination rate for the third dose was different from those of the first two doses because the government allowed those recovered from COVID an exemption from the third jab requirement. The vaccination rates for the first and third doses reached the maximum during the outbreak of the fifth wave.

The coefficient of determination was calculated, and it showed the time difference between the first two consecutive doses. A Large time delay (35 days, *R*^2^ = 0.9999) and vaccine hesitancy were observed in people who received the CoronaVac vaccine. For BNT162b2 (23 days, *R*^2^ = 0.9994), the result was aligned with the government’s suggestion of the time interval between the first and second doses. According to the Centre for Health Protection (CHP), extending the interval between COVID-19 vaccine doses can increase immune responses and reduce the risk of adverse effects such as myocarditis, particularly in younger males. These recommendations likely influenced individuals to delay their second dose in hopes of achieving better immunity and fewer side effects. Research has shown that longer intervals between doses lead to higher concentrations of neutralizing antibodies, improving overall vaccine effectiveness (10, 15). The time difference between the overall vaccination rate for the first two doses deviated from the suggestion of the CHP. This deviation can be attributed to various factors including public fears of adverse effects, government restrictions, and the intention to gain a stronger immune response. Recovery from COVID-19 in the early 2022 was considered one dose of vaccination. Thus, those who recovered were given a six-month immunity period to receive the subsequence dose.

### Analysis 2

Supplementary Figure S2 shows the vaccine options, with most people preferring BNT162b2 for the first two doses. For the third dose onwards, more people opted to receive a different vaccine, as combining the two types of vaccines may have provided the immune system with multiple ways to recognize a pathogen. Thus, they achieved stronger immunity.

According to CHP bulletins and data from vaccine manufacturers, periodic shortages and distribution challenges affected the availability of both vaccines, leading to fluctuations in vaccine preferences (10). This highlights that the observed trends in vaccine uptake may not solely reflect population demands and preferences but also the logistical aspects of vaccine distribution.

The CHP provided only the data on the number of people who received the vaccines, necessary for investigating the vaccination rates across different age groups. Therefore, the population data for these age groups can be found at https://www.coronavirus.gov.hk

Supplementary Figure S3 illustrates the age-specific preferences for vaccination dose and brand. The preferences were similar for both the first and second doses. In the 0–11 age group, for the first dose, 77% chose CoronaVac, while 23% opted for BNT162b2, reflecting the government’s recommendation of CoronaVac for younger children. Starting from the third dose, the preference among the older adult shifted toward BNT162b2, whereas younger individuals (aged 0–11 and 12–19) continued receiving CoronaVac, thereby gaining the benefits of both vaccines. The majority in the 12–59 age group preferred BNT162b2, but those in the 0–11 and 60 or above age groups favored CoronaVac, primarily due to its fewer side effects (16, 17).

### Analysis 3

The launch of the Comirnaty Original/Omicron BA.4/BA.5 bivalent vaccine on December 1, 2022, encouraged more people to take the new vaccine.

Table 1 shows vaccination hesitancy due to reservations about the existing brands of vaccines. The Comirnaty Original/Omicron BA.4/BA.5 bivalent vaccine more effectively protected against the Omicron variant, contrary to existing BNT162b2 vaccines. This new bivalent vaccine encouraged more people from all age groups to take the new vaccines. The Poisson regression for counts show a significant increase in the number of Comirnaty Original/Omicron BA.4/BA.5 bivalent vaccinations for all age groups except 12–19 (*p*-value = 0.3947) and 80 or above (*p*-value = 0.8203). Vaccination hesitancy could have been minimized by introducing new vaccines, such as the Comirnaty Original/Omicron BA.4/BA.5 bivalent vaccine in this example. Besides the launch of new vaccines, the number of infected cases from December 1–7 (71,172 cases) was 20.32% higher than that of November 24–30 (59,154 cases). People were more aware of their health when the pandemic worsened. These data were obtained from the Centre for Health Protection’s weekly reports on COVID-19 cases (18).

**Table 1:** Mean, standard deviation of weekly vaccination dose intake and result of poisson regression for count.

Age group	CoronaVac (24–30 Nov)		CoronaVac (1–7 Dec)		*p*-value	BNT162b2 (24–30 Nov)		Comirnaty Original/Omicron BA.4/BA.5 bivalent (1–7 Dec)		*p*-value
	Mean	*SD*	Mean	*SD*		Mean	*SD*	Mean	*SD*	
12–19	132.29	43.95	145.43	56.07	ns	214.71	101.18	197.43	77.46	ns
20–39	93.57	29.26	123.29	35.04	< 0.05	232.86	58.77	293.43	36.05	< 0.01
30–39	170.57	46.51	195.29	52.89	ns	260.00	53.32	318.71	71.27	< 0.05
40–49	175.71	44.73	184.71	54.55	ns	144.57	24.57	195.43	37.04	< 0.01
50–59	212.86	62.05	227.86	67.71	ns	132.71	31.31	176.43	39.61	< 0.05
60–69	296.29	121.05	305.86	107.34	ns	118.86	33.93	168.14	45.70	< 0.01
70–79	276.29	124.22	258.71	95.81	ns	69.43	26.54	90.00	28.97	ns
80 or above	289.57	128.12	254.14	92.43	ns	43.43	14.25	45.57	18.82	ns
Overall	1647.14	555.74	1695.29	483.89	ns	1216.57	259.90	1485.14	264.32	< 0.01

### Analysis 4

The top rows of Supplementary Figure S4 and Table 2B show that the fatality rate of the unvaccinated group (14.32%) of 80 years or above was twofold to those who received one dose (7.11%). The fatality rate of those who received BNT162b2 was generally lower than those who received CoronaVac. Three doses could have reduced the fatality rate of people 80 years or above from 14.32% to 2.27%.

**Table 2:** (A) Number of fatalities. (B) Fatality rate by age group, vaccination status and vaccines since the fifth Wave (as of January 4, 2023, CHP reported the statistics on every Wednesday).

A											
Age group	<3	3–11	12–19	20–29	30–39	40–49	50–59	60–69	70–79	80+	*p*-value
Grand total	4	8	6	19	31	84	342	1,010	1,949	8,337	
Unvaccinated	4	5	3	9	16	44	170	555	1,123	5,373	
1 Dose	0	2	1	1	4	6	34	131	273	1,110	
CoronaVac	0	1	0	1	2	6	26	104	245	1,028	< 0.0001
BNT162b2	0	1	1	0	2	0	8	27	28	82	
2 Dose	0	1	1	9	4	21	86	189	298	929	
CoronaVac	0	1	0	3	0	8	54	132	235	819	< 0.0001
BNT162b2	0	0	1	6	4	13	31	57	63	109	
3 Dose	0	0	1	0	8	15	53	134	233	844	
CoronaVac	0	0	1	0	3	8	29	92	175	728	< 0.0001
BNT162b2	0	0	0	0	4	5	22	31	47	89	
4 Dose	0	0	0	0	0	0	2	14	38	116	
CoronaVac	0	0	0	0	0	0	0	4	24	96	< 0.0001
BNT162b2	0	0	0	0	0	0	2	8	9	13	

B										
Age group	<3	3–11	12–19	20–29	30–39	40–49	50–59	60–69	70–79	80+
Grand total	0.0001	0	0	0.0001	0.0001	0.0002	0.0008	0.0028	0.0109	0.0686
Unvaccinated	0.0001	0.0001	0.0003	0.0003	0.0003	0.0013	0.0059	0.0157	0.0426	0.1432
1 Dose	0	0.0001	0.0001	0.0001	0.0002	0.0004	0.0020	0.0053	0.0153	0.0711
CoronaVac	0	0.0001	0	0.0003	0.0003	0.0007	0.0022	0.0055	0.0163	0.0725
BNT162b2	0	0.0003	0.0001	0	0.0002	0	0.0016	0.0048	0.0098	0.0576
2 Dose	0	0	0	0.0001	0	0.0002	0.0007	0.0017	0.0065	0.0388
CoronaVac	0	0	0	0.0002	0	0.0002	0.0008	0.0020	0.0076	0.0429
BNT162b2	0	0	0	0.0001	0	0.0002	0.0005	0.0013	0.0043	0.0226
3 Dose	0	0	0	0	0	0.0001	0.0002	0.0008	0.0032	0.0227
CoronaVac	0	0	0.0001	0	0.0001	0.0001	0.0003	0.0011	0.004	0.0257
BNT162b2	0	0	0	0	0	0	0.0002	0.0005	0.0021	0.0129
4 Dose	0	0	0	0	0	0	0.0001	0.0004	0.0022	0.0150
CoronaVac	0	0	0	0	0	0	0	0.0003	0.0024	0.0174
BNT162b2	0	0	0	0	0	0	0.0003	0.0008	0.0020	0.0095

From Table 2A, sufficient evidence suggested an association between fatality and vaccine types (*p*-value <0.0001). It is important to consider that individuals in relatively poorer health conditions are less likely to opt for vaccines with higher side effects. This aligns with the CHP recommendations from September 15, 2021, which emphasize that older adults with acute illnesses should carefully choose their vaccines based on side effects profiles. The lower fatality rates observed in those who received BNT162b2 could be partially attributed to healthier individuals selecting this vaccine due to its higher efficacy despite its higher incidence of side effects compared to CoronaVac (19).

### Analysis 5

Supplementary Figure S5 shows the relationship between the basic reproduction number and herd immunity under different SARS-CoV-2 variants. The figure shows that the minimum threshold of herd immunity for the Omicron variant, BF.7 was 90% (20).

Supplementary Figure S6 indicates that when three doses are required to achieve the effective dosage, none of the age groups has reached herd immunity. It is important to factor in the infected individuals who did not need to receive the vaccine for the next 3 to 9 months after infection, depending on their general immune competence. Given that Omicron had a basic reproduction number (BRN) of 90%, it means that most of the population would not require vaccines until around June to September 2022. This situation skewed the vaccination data toward the negative axis, as many individuals relied on natural immunity during this period rather than receiving additional vaccine doses (21–23).

Table 3 shows that vaccination hesitancy was serious for younger age groups (0–11). Approximately 31% of that age group was willing to receive the third dose. It is important to note that the general supply and availability of vaccines, as well as the timelines for adopting vaccines for ages 0–3 years, likely influenced this hesitancy. The CHP gradually rolled out vaccinations for the younger population and recommended that pediatric vaccines be improvised by diluting adult doses. These factors, along with potential skepticism about the safety and efficacy of these improvised vaccines, may have contributed to the observed hesitancy. The government should have allocated more resources to improve the vaccination rate for school children. For example, it would have been recommended that the Department of Health collaborate with Education Bureau to have provided more scientific information, educational and on-site vaccination interventions to schools with low vaccination rates (24), enhance the biological literacy of the school children to have enhanced their willingness to vaccinate (25), ensure that health professions respond to rumors and conspiracy theories immediately (26), explain and communicate the potential benefits of vaccination to parents and resolve vaccination hesitancy via online education seminar (27, 28). A study also found that continued regular education resulted in vaccination success (29). Priority of vaccination also should have been given to people aged 80 years or above, as their vaccination rate is low (63.67%) and fatality rate high. Supplementary Figure S7 displays the top five age groups demonstrating vaccine hesitancy, which include those aged 70–79, 80 and above, 60–69, 50–59, and 0–11.

**Table 3:** Vaccination rate (%) for the first three doses by age group.

Age group	First dose overall	CoronaVac (count)	BNT162b2 (count)	Second dose overall	CoronaVac (count)	BNT162b2 (count)	Third dose overall	CoronaVac (count)	BNT162b2 (count)
0–11	93.14	361,569	106,573	81.39	327,220	81,863	31.05	154,066	2,009
12–19	101.03	69,564	382,335	99.70	72,791	373,171	77.05	82,743	261,882
20–29	99.62	145,104	623,889	98.01	142,016	614,525	79.56	108,517	505,609
30–39	100.95	303,911	800,993	99.74	297,861	793,827	83.34	218,631	693,481
40–49	100.97	471,691	699,813	100.36	466,891	697,618	89.68	350,123	690,467
50–59	97.48	583,657	577,631	96.86	578,734	575,142	88.99	446,018	614,104
60–69	89.34	591,101	411,355	88.80	586,047	410,357	83.92	476,067	465,622
70–79	83.43	332,668	160,659	83.10	330,781	160,573	80.45	289,378	186,340
80 or above	71.87	228,564	57,607	69.85	221,525	56,606	63.67	191,829	61,687
All	94.91	3,087,829	3,820,855	93.24	3,023,866	3,763,682	79.66	2,317,372	3,481,201

In addition to offering BNT162b2 and CoronaVac, the government could have considered offering fractional doses to the age group with low vaccination rates to have ensured they are vaccinated with minimizing side effects (30–32). Also, another study (33) suggested that an intradermal fractional dose (0.1–0.2 amount of the original dose) vaccination, administered using a microneedle patch, could have produce results of better immunogenicity.

Supplementary Figure S8 shows a time series forecast to predict the vaccination rate using the SPSS expert modeler. The finding suggests that ARIMA (6,2,2) is the best model, and achieving herd immunity solely by vaccinating 90% of the population was impossible. If a person fully recovered from COVID-19 was considered to have taken one dose, then given the 2022 circumstance, reopening the border and relaxing social measures were viable considerations as the pandemic was ending. The government should have adjusted the vaccination strategy after March 2023 for better allocation of resources. Moreover, the daily COVID-19 re-infection rate in Hong Kong was approximately 1% and this figure was underestimated due to the emergence of the Omicron variant, and how vaccination could have only minimized the risk of symptomatic and asymptomatic infection. The antibody levels of vaccinated groups had gradually declined. Booster immunisations were recommended and provided to high-risk groups, such as healthcare workers and patients with comorbidities, to achieve long-term control of COVID-19 and avoid extra morbidity and death (34). The Centre for Health Protection (CHP) had issued consensus interim recommendations on September 15, 2021, emphasizing the importance of booster doses for these high-risk populations (19).

The nonseasonal ARIMA model introduced by Box and Jenkins (35) shows the various model structures with the notations ARIMA (*p*, *d* and *q*).

#### Model identification

The time-series analysis was performed using the ARIMA model to predict the time required to achieve herd immunity. The model identification process began with determining the appropriate order of differencing (*d*) required to make the series stationary. Autocorrelation Function (ACF) and Partial Autocorrelation Function (PACF) plots were analyzed to identify the potential autoregressive (*p*) and moving average (*q*) terms. The Expert Modeler feature in IBM SPSS software was used to automatically identify and select the best-fitting model based on these criteria.

Schwarz (36) developed the Bayesian Information Criterion for model selection (BIC). Selection criteria, such as R-square, stationary R-square, root mean square error (RMSE), mean absolute percentage error (MAPE) and BIC are used in Supplementary Table S2 to determine which ARIMA model fits the data the best.

#### Parameter estimates

Once the model was identified, the parameters for the ARIMA (*p*, *d*, *q*) model were estimated using maximum likelihood estimation within SPSS. The software provided estimates for the autoregressive coefficients (*p*), the degree of differencing (*d*), and the moving average coefficients (*q*). These parameters were selected based on their statistical significance and their ability to minimize the information criteria values (AIC and BIC).

To fit the ARIMA model, we first examined the autocorrelation function (ACF) and partial correlation function (PACF) in Supplementary Figure S9 to determine the stationary or nonstationary status of the time series. The significance of the autocorrelation coefficients was assessed using the Ljung-Box Chi-Square test (Q-test). We then selected the AR terms based on the PACF plot, ensuring a larger view to identify the cutoff point for AR terms selection. The initial fitting showed a perfect R-squared value of 1.000, indicating potential overfitting. To address this, we refitted the ARIMA model with higher order AR terms as suggested by the PACF plot, which reduced the R-squared value and improved the fit statistics, ensuring a more statistically adequate description of the time series.

A time series’ stationary or nonstationary status can be determined using the autocorrelation function (ACF) and partial correlation function (PACF) in Supplementary Figure S9, and the significance of the autocorrelation coefficients can be determined using the Ljung-Box Chi-Square test (*Q*-test).

### Diagnostic checking

After estimating the model parameters, diagnostic checks were performed to validate the model. The residuals of the fitted ARIMA model were examined using the ACF and PACF plots to ensure that they behaved like white noise, indicating that the model adequately captured the underlying data patterns. The Ljung-Box *Q* test was also applied to test the overall randomness of the residuals, ensuring no significant autocorrelation remained.

Several methods can be used to assess if the selected model provides a statistically adequate description of the time series.

Once the time-series stationary has been achieved, the identification model process, which makes use of information on how time-series are created, starts. The goal is to gain a basic notion of the values of *p*, *d* and *q* in the ARIMA general linear model prior to determining some rough estimations of the model parameters.

#### Forecasting

With the model validated, it was then used to generate forecasts for the vaccination rates and predict the time required to achieve herd immunity. The ARIMA model’s forecast accuracy was evaluated by comparing the predicted values with the actual values during a holdout sample period. The model’s performance was assessed using fit statistics, including Mean Absolute Percentage Error (MAPE) and Root Mean Square Error (RMSE).

Supplementary Table S3 shows that the actual values ^1^and predicted values of the vaccination rates were similar. However, during the Chinese New Year (21st, 22nd, 24th, 25th and 27th of January), the actual vaccination rates did not increase remarkably. Traditional Chinese Taboos prohibited people from taking medications or visiting doctors before the Lantern Festival, as doing so could have left them unwell all year long with little chance of recovery. This traditional belief could have been the explanation to the vaccination hesitancy over the Chinese New Year.

## Discussions

The vaccination program led to more vaccinations, having a longer gap between the first and second doses for CoronaVac than BNT162b2, concordant with the government’s suggestion but with a slight deviation. A more significant proportion of the population aged 50 and above preferred CoronaVac over BNT162b2. Thus, the longer days between the first two doses of CoronaVac could be due to skepticism from the older generations. In addition, more people vaccinate when a spike in the number of infected cases occurs. Moreover, those who recovered from COVID-19 infections may opt not to take the third vaccination for 6 months, reflected by the decrease and lag in the third dose vaccinations. However, the effect of imported cases on vaccine hesitancy cannot be deduced due to insufficient information.

Apart from the average time between the first two doses, a shift in the preferred vaccine brands for the third dose is observed, where the older adult preferred BNT162b2 and the younger people received CoronaVac, due to the stronger immunity from mixing vaccines. Studies have shown that mixing COVID-19 vaccines, such as combining an mRNA vaccine (e.g., BNT162b2) with an inactivated vaccine (e.g., CoronaVac), can enhance immunogenicity. These mixed regimens have been found to elicit higher antibody responses and stronger cellular immunity compared to homologous booster doses (37, 38). Although most children aged 0–11 and older adult 80 years or above still preferred CoronaVac perhaps due to the milder side effects. For children aged 0–11, the vaccination decisions were made by their parents or guardians, as children in Hong Kong are not allowed to provide consent for themselves.

Furthermore, the launch of new vaccines reduced vaccine hesitancy given that they are more effective at targeting the newer variants of the virus. Apart from the age groups 0–11 and 80 above, a significant increase in the number of vaccinations is observed. However, such an increase in vaccinations does not affect the increase in infected cases. Therefore, the reduction in vaccine hesitancy is probably mostly due to the newer vaccines, but not entirely. An important factor contributing to this reduction is the fear of getting infected or becoming seriously ill. People are more likely to comply with vaccination when they are concerned about the potential health risks posed by COVID-19. This heightened awareness and concern for personal health have played a significant role in encouraging vaccination uptake.

Vaccines significantly reduce the mortality rate, especially for those aged 80 or above, and BNT162b2’s vaccines lead to a lower mortality rate than CoronaVac’s. However, it is important to consider the potential selection bias in these results, as individuals with acute illnesses were often excluded from vaccination due to concerns about side effects. This exclusion may have affected the observed mortality rates, as healthier individuals were more likely to receive the vaccines. The Centre for Health Protection (CHP) emphasized the careful selection of vaccines for older adults with acute illnesses in their September 15, 2021 (19). This selection bias highlights the need to interpret the mortality data with caution and consider the health status of the vaccinated individuals. Although studies have shown that BNT162b2’s vaccines have a higher probability of developing complications, which are exacerbated in infants and the older adult (39, 40). It is important to note that only relatively healthy adults and children are more likely to risk higher side effects, as individuals with acute illnesses were often excluded from vaccination due to concerns about side effects. This selection bias must be considered when interpreting the safety and efficacy of the vaccines. The Centre for Health Protection (CHP) has issued numerous bulletins emphasizing careful vaccine selection for those with underlying health conditions (41).

Despite the vaccines’ effectiveness, vaccine hesitancy results in a lack of herd immunity, which requires 90% or more vaccinated individuals in a population. For example, less than one-third of younger children aged 0–11 is vaccinated. In addition, the vaccination rate is unlikely to increase dramatically to overcome the 90% mark when the data are extrapolated. This prediction indicates that achieving herd immunity through vaccination alone may be challenging. However, natural immunity acquired through previous infections also contributes to overall immunity in the population, which aids in COVID-19 recovery. In addition, due to various traditions, such as not taking medicine before the Lantern Festival, vaccine hesitancy can occur at specific time periods when visiting vaccination centers is unpopular.

While this study has provided valuable insights into vaccine hesitancy and its impact on achieving herd immunity, it is important to acknowledge the limitations regarding the alignment of our findings with existing literature. Direct comparisons with existing studies are limited, which highlights the unique contributions of our research. The detailed analysis of age-specific vaccine hesitancy and the impact of bivalent vaccines are novel aspects of this study. Future research should aim to validate our findings by comparing them with data from other regions and populations, thereby providing a broader context for interpretation. Additionally, longitudinal studies could further elucidate the long-term effects of vaccination strategies on herd immunity and public health outcomes.

## Limitations

This study, while comprehensive in its approach, is subject to several limitations. Firstly, being a secondary data analysis, it relies on the accuracy and completeness of data from official databases, which may have inherent biases or gaps. Additionally, the focus on Hong Kong means the findings may not be generalizable to other regions with different demographic, cultural, and healthcare contexts. The analysis also does not account for real-time changes in vaccine efficacy or emerging COVID-19 variants, which could affect the applicability of the results over time. Furthermore, the statistical methods, although robust, have limitations in capturing the complexities of human behavior and decision-making processes in vaccine hesitancy.

However, this study also possesses several strengths. It utilizes an extensive dataset spanning over 1,000 days, providing a robust longitudinal perspective on COVID-19 vaccination and hesitancy trends. The use of sophisticated statistical methods, including time-series analysis and ARIMA modeling, enhances the reliability of the predictions and conclusions drawn. Additionally, the study’s focus on vaccine hesitancy across different age groups provides valuable insights that can inform targeted public health interventions. These strengths contribute to the study’s potential to guide policymakers in improving vaccination strategies and addressing hesitancy effectively.

## Implications

The findings of this study have significant implications for public health policy and vaccination strategies. By identifying key factors contributing to vaccine hesitancy, health authorities can tailor their communication and outreach efforts to address specific concerns and misconceptions. The importance of targeted vaccination campaigns for age groups with higher hesitancy is highlighted, suggesting that personalized approaches may be more effective. The impact of bivalent vaccines on reducing hesitancy and improving fatality rates underscores the need for ongoing vaccine development and public education about vaccine benefits. Moreover, the insights gained from this research can assist policymakers in devising strategies to increase vaccination uptake, which is crucial for achieving herd immunity and controlling the pandemic.

## Conclusion

In conclusion, vaccine hesitancy is an obstacle that needs to be overcome to achieve the final goal of herd immunity. This can be addressed by carefully publishing information on the increase in COVID-19 cases to create a sense of urgency without causing public panic, introducing updated vaccines, and prioritizing vaccinations for those more susceptible to COVID-19. Clear and accurate communication is essential to ensure the public understands the importance of vaccination without inducing fear. Although the recent strains of COVID-19 tend to be weaker, children and the older adult should vaccinate more given that only a low proportion of their population has received the vaccine. It is also important to note that while environmental monitoring of wastewater and surface water is crucial, there is currently no evidence of a direct human health risk from exposure to virus-contaminated environmental samples. From Table 2, the relative fatality rate among children is significantly lower than that of the older adult. However, the decision to vaccinate children should consider both the direct benefits of preventing severe illness and the broader public health benefits of reducing transmission. While the risks of severe outcomes from COVID-19 are generally lower in children, vaccination can help protect vulnerable populations and contribute to overall herd immunity. Careful consideration of the risks and benefits, along with consultation with healthcare professionals, is essential in making vaccination decisions for children. CoronaVac leads to fewer side effects and could be considered for such priority groups that aim to minimize side effects, but if side effect risks are deemed low, BNT162b2 can be considered because it leads to fewer fatalities. The governments could relax COVID-19 control measures. However, vaccinations should continue to increase the chance of herd immunity when a random individual becomes more susceptible to this virus.

## Data Availability

The raw data supporting the conclusions of this article will be made available by the authors, without undue reservation.

## Footnotes

^1^adopted from: https://www.coronavirus.gov.hk/
