# Supplementary material for: Predicting herd immunity achievement: a time-series analysis of vaccination and fatality rates using 1,075 days of COVID-19 data
Source: Front Public Health. 2024 Sep 20;12:1403163. doi: 10.3389/fpubh.2024.1403163 (PMC11449892; doi:10.3389/fpubh.2024.1403163)
Supplement: Supplementary file 1 [file Data_Sheet_1.pdf]

Supplementary Materials of the paper titled

Predicting Herd Immunity Achievement: A Time-Series Analysis of Vaccination and Fatality Rates Using 1075 Days of COVID-19 Data

[1] Department of Mathematics, The Chinese University of Hong Kong, Sha Tin, Hong Kong SAR, China,

[2] Department of Psychology, University of Science and Technology of China, Hefei, China

[3] King George V School, Ho Man Tin, Hong Kong SAR, China

[4] Department of Diagnostic Radiology, Li Ka Shing Faculty of Medicine, The University of Hong Kong, Pok Fu Lam, Hong Kong SAR, China

[5] School of Nursing, Tung Wah College, Ho Man Tin, Hong Kong SAR, China

[1,2, 3]co-first authors

Benny Yiu Chung Hon[1,2],

benny.hon@math.cuhk.edu.hk

Jeffrey Chan[3],

chanjn3@kgv.hk

Kei Shing Ng[4],

dougng@hku.hk

Corresponding Author:

Simon Ching Lam[5*]

PhD, RN, FHKAN, FAAN

Associate Dean and Professor,

School of Nursing, Tung Wah College,

Email: simlc@alumni.cuhk.net; simonlam@twc.edu.hk

ORCID: 0000-0002-2982-9192

Phone: 852 34686854

Supplementary Material A: Statistical Methods

1. **ARIMA Models:** The AutoRegressive Integrated Moving Average (ARIMA) models, introduced by Box and Jenkins, were used to predict vaccination trends. The model parameters (p, d, q) were selected based on the Partial Autocorrelation Function (PACF) and Autocorrelation Function (ACF) plots. Model fit was evaluated using criteria such as R-squared, stationary R-squared, Root Mean Square Error (RMSE), Mean Absolute Percentage Error (MAPE), and Bayesian Information Criterion (BIC).
2. **Hypothesis Testing:** Various hypothesis tests were conducted to determine the significance of the results. The Ljung-Box Chi-Square test (Q-test) was used to test for autocorrelation in the residuals of the fitted ARIMA models, ensuring that the model was adequate.
3. **Assumptions:** The primary assumptions for the ARIMA model include stationarity, invertibility, and the normality of residuals. Stationarity was checked using the ACF and PACF plots, and differencing was applied if needed to achieve stationarity. Residual analysis was performed to ensure that the residuals of the models followed a normal distribution.
4. **Data Cleaning and Preparation:** The data underwent rigorous cleaning and preparation. This included handling missing values, removing duplicates, and ensuring consistency in the data format. Outliers were identified and addressed appropriately to prevent skewed results.

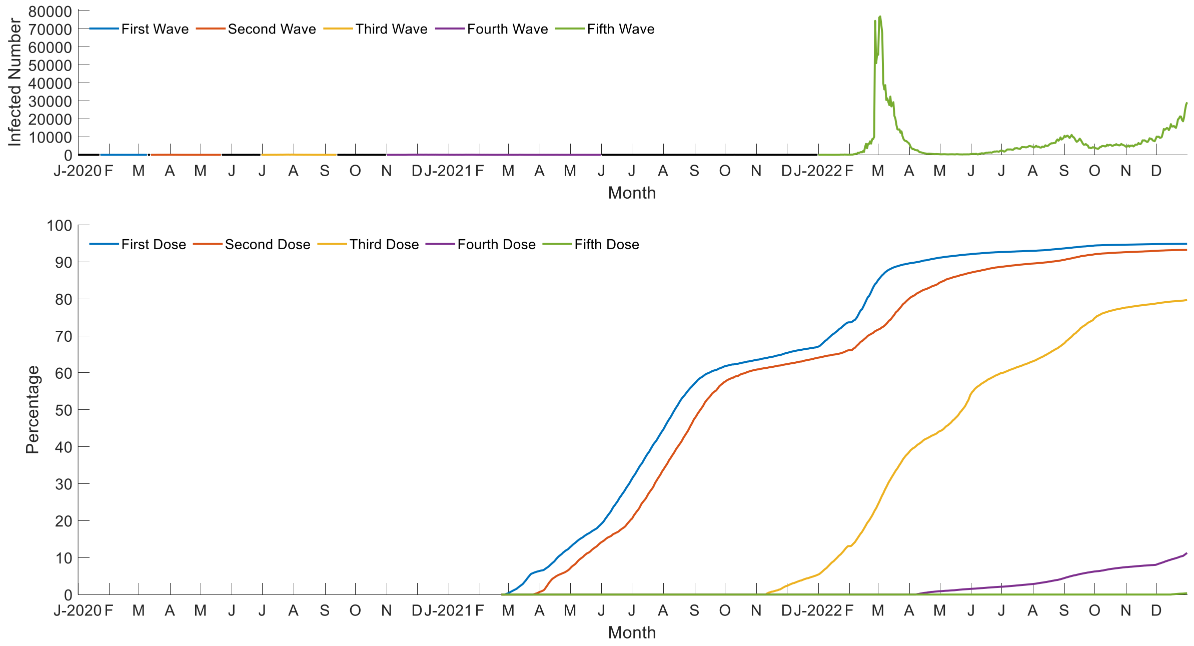

Figure S1: Daily infected cases and vaccination rate for different age groups between the 22nd of February 2021 and the 31st of December 2022.

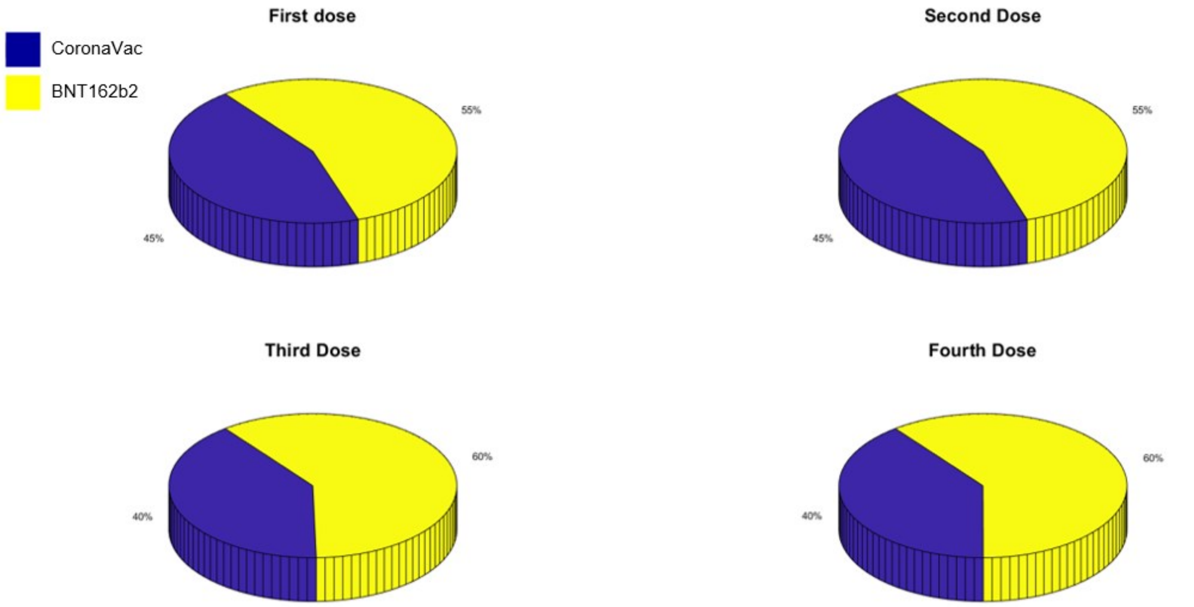

Figure S2: Preference of vaccine brands

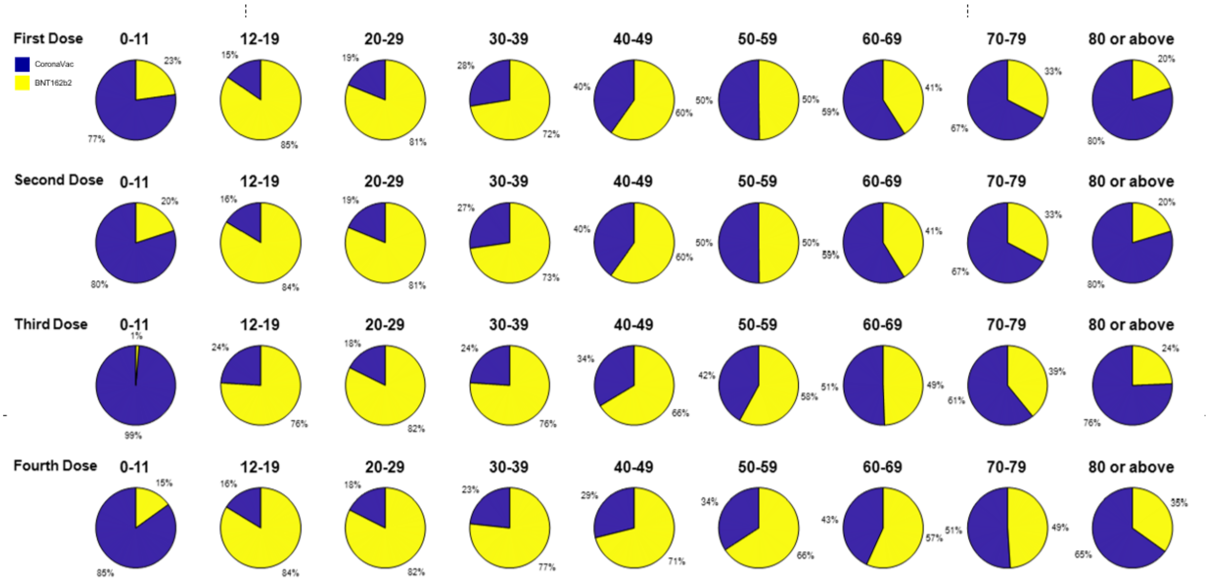

Figure S3: Age specific preference on the vaccination dose and brand.

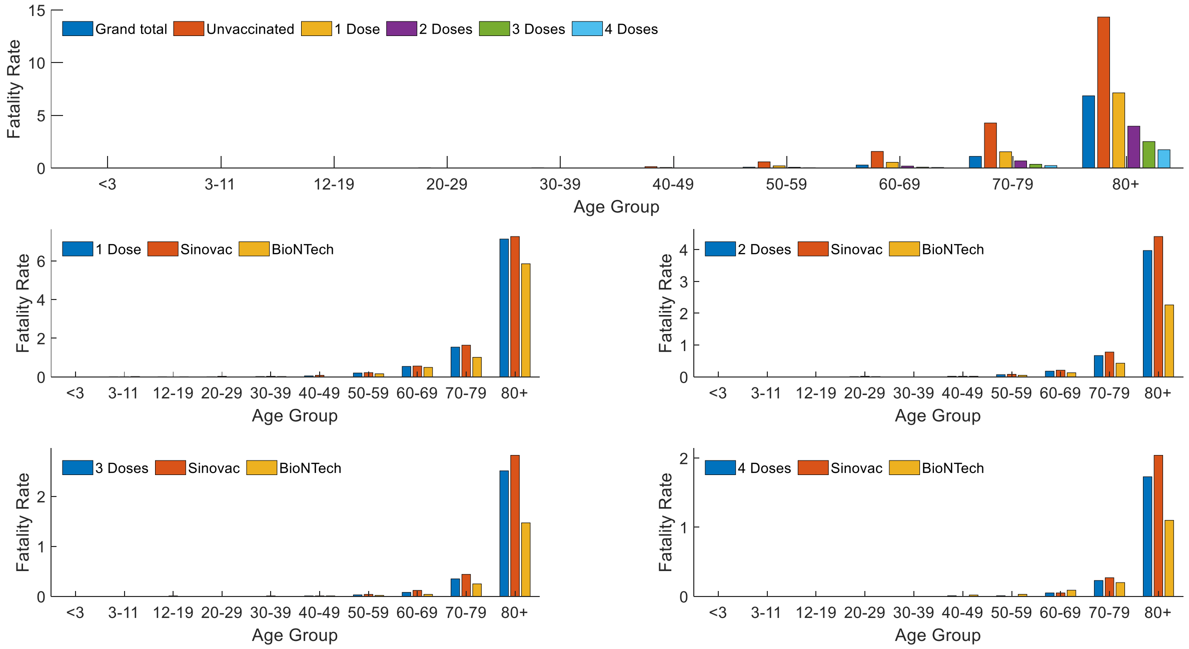

Figure S4: Case fatality rate by age group and vaccination status

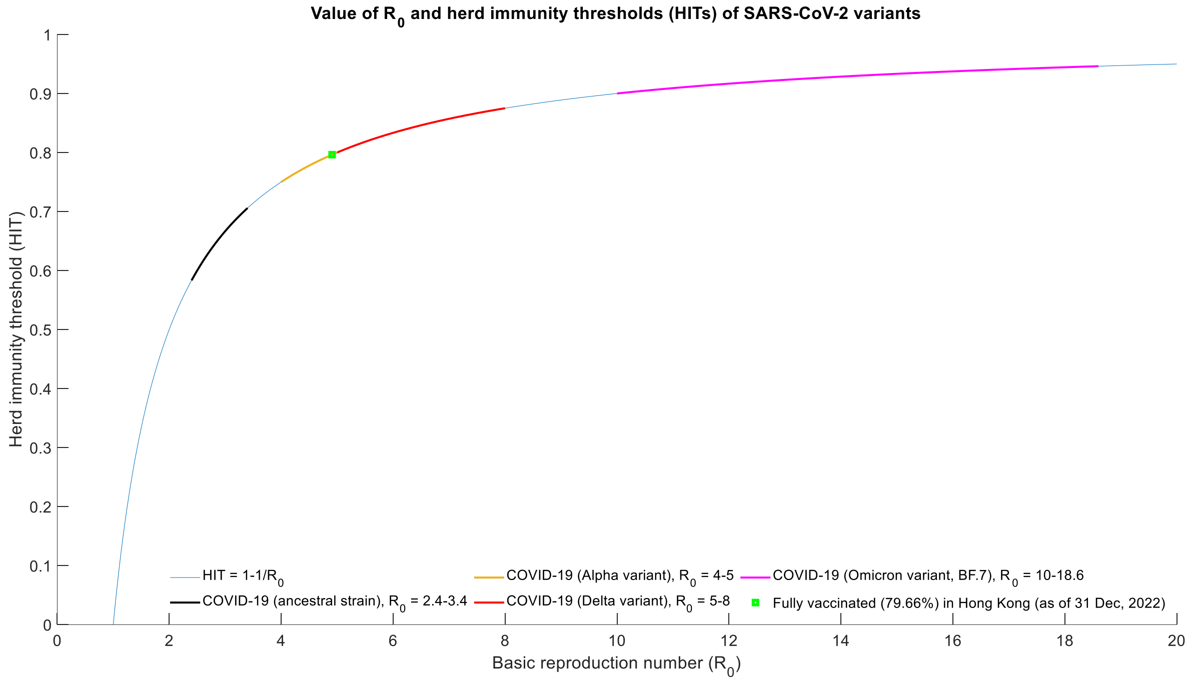

Figure S5: Relationship between the basic reproduction number and herd immunity. For example, if the reproduction number is 10, then the minimum herd immunity is 90%.

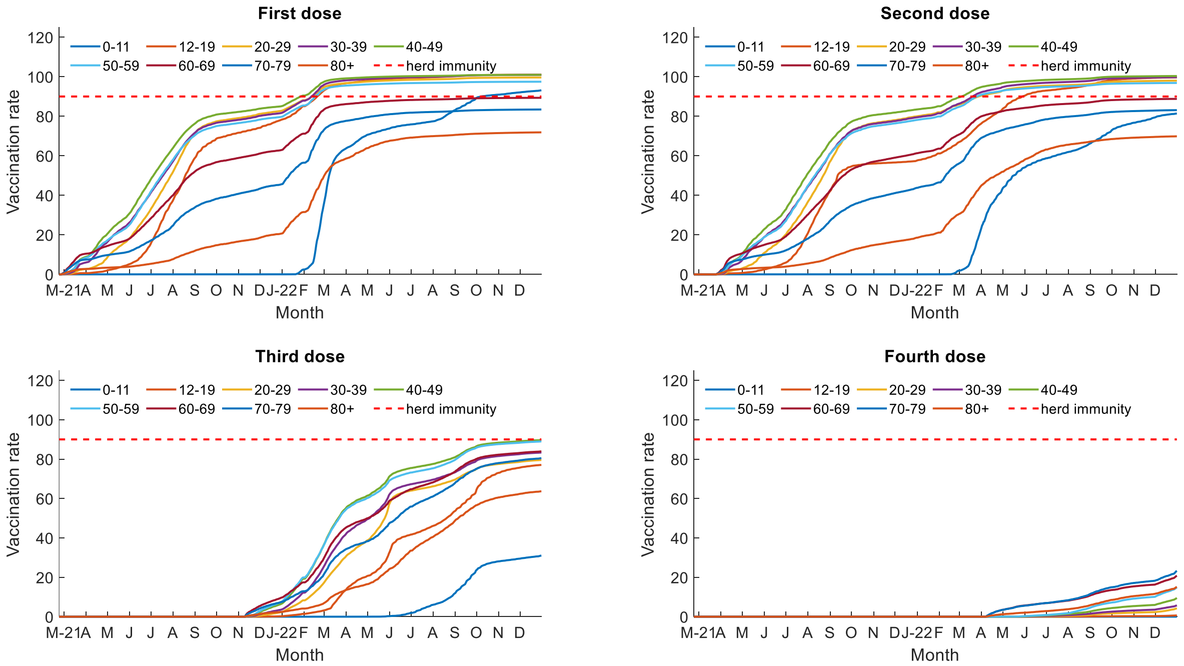

Figure S6: Vaccination rates of non-Hong Kong residents (such as holders of the Exit-Entry Permit for travelling to and from Hong Kong and Macao with two complete doses of vaccination within their stay limit without extension, nonrefoulement claimants and refugees and other visitors staying in Hong Kong). Vaccination rates may be greater than 100% on the graph as it includes visitors for some age. Thus, when the total population of Hong Kong residents exceeded the original population, a value greater than 100% is obtained.

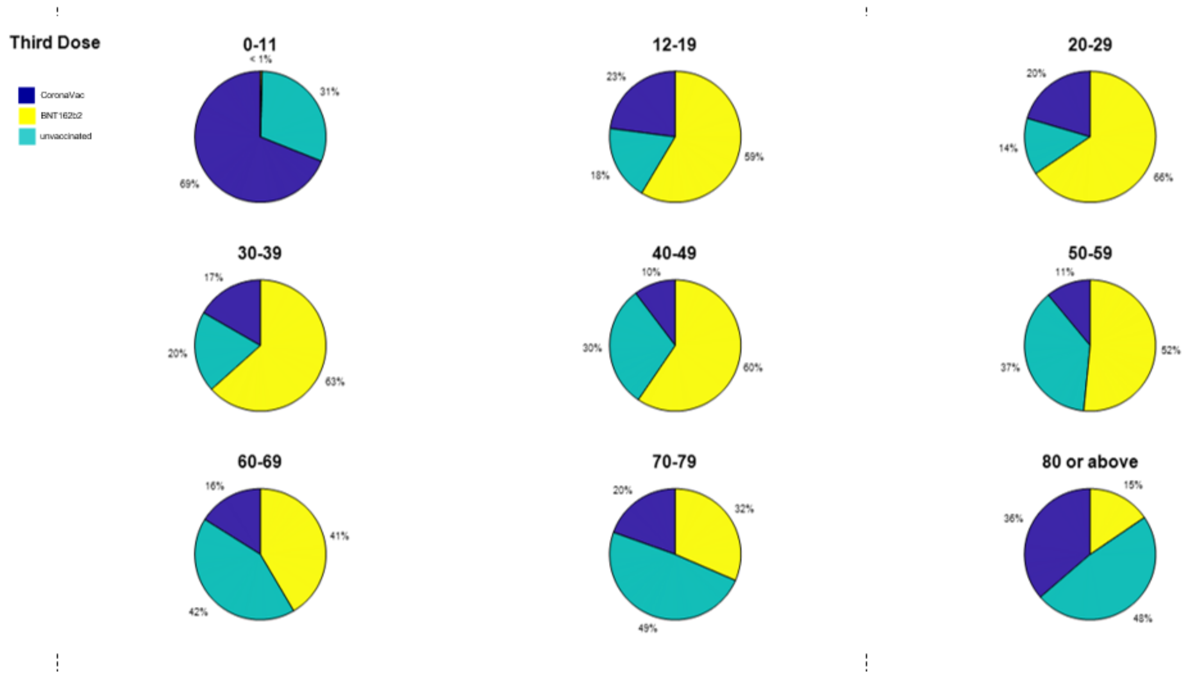

Figure S7: Top five age groups with vaccination hesitancy include 70–79, 80 or above, 60–69, 50–59 and 0–11.

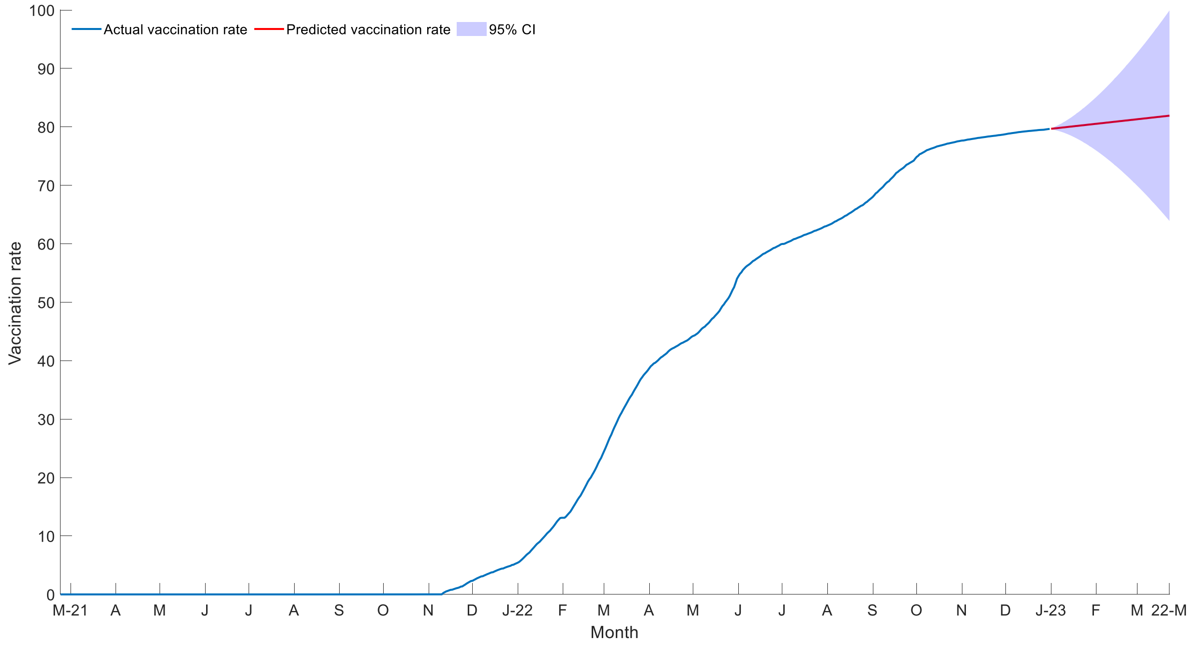

Figure S8: Time series forecast to predict the vaccination rate using SPSS Expert Modeler. The vaccination rate for the third dose is expected to reach 81.92% on March 22, 2023.

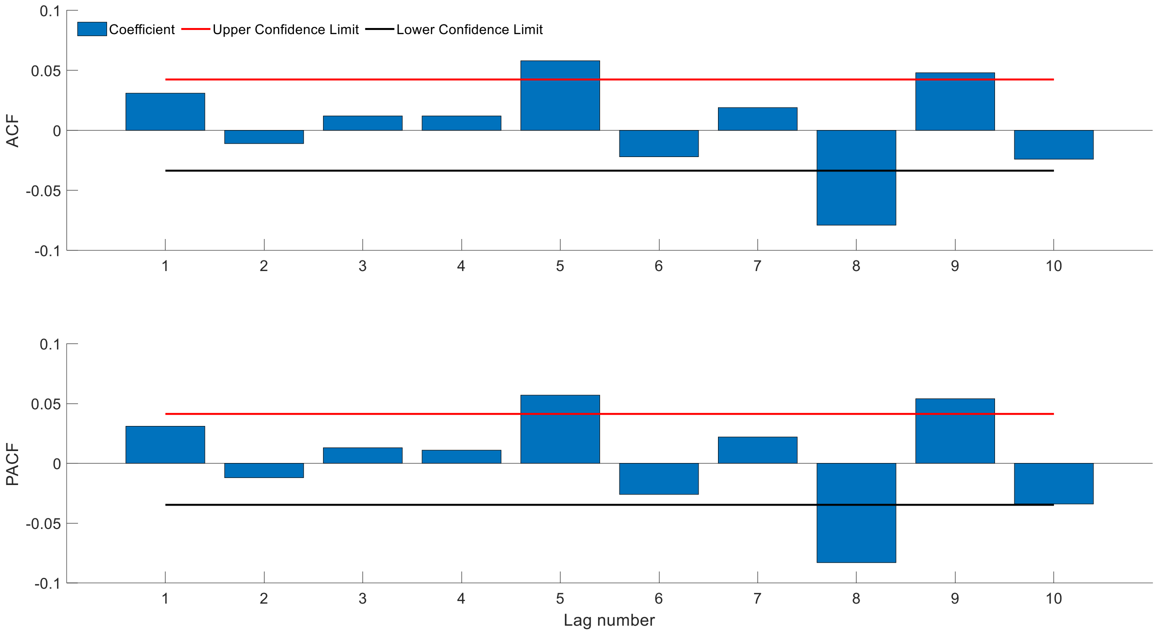

Figure S9: ACF and PACF plots of cumulative percentage

Table S1: Number of infected. death cases and its dominant variants in Hong Kong

Wave	Variants	Days	Infected*	Death*	Death rate# (%)
First	-	41	< 100	2	2.00
Second	-	33	800+	2	0.25
Third	Beta	75	3,700+	93	2.50
Fourth	Alpha, Beta, Delta, Gamma	184	5,700+	102	1.79
Fifth	Delta, Omicron	365+	2.61M+	11,000+	0.44

* Provisional figures, #approximated values. (Adpoted from: https://data.gov.hk/en-data/dataset/hk-dh-chpsebcddr-novel-infectious-agent)

Table S2: ARIMA model Description

Model Statistics													
Model	Number of predictors	Model Fit Statistics								Ljung-Box Q(18)			Number of Outliers
		Stationary R-squared	R-squared	RMSE	MAPE	MAE	MaxAPE	MaxAE	Normalized BIC	Statistics	DF	Sig.	
Cumulative Percentage_Model_1	0	.338	1.000	.000	1.172	.000	100.000	.003	-15.675	31.278	10	.001	0

Table S3: Actual value (first row) vs. predicted value (second row) of the cumulative vaccination rate of the third dose on January 1–29, 2023.

Sunday	Monday	Tuesday	Wednesday	Thursday	Friday	Saturday
1	*2*	*3*	*4*	*5*	*6*	*7*
79.67<br>79.68	79.69<br>79.70	79.71<br>79.73	79.74<br>79.76	79.77<br>79.78	79.80<br>79.81	79.83<br>79.84
8	*9*	*10*	*11*	*12*	*13*	*14*
79.85<br>79.87	79.87<br>79.90	79.89<br>79.92	79.91<br>79.95	79.93<br>79.98	79.96<br>80.01	79.98<br>80.04
15	16	17	18	19	20	21
79.99<br>80.06	80.00<br>80.09	80.02<br>80.12	80.03<br>80.15	80.04<br>80.17	80.05<br>80.20	80.05<br>80.23
22	***23***	***24***	***25***	*26*	*27*	*28*
80.05<br>80.26	80.06<br>80.28	80.06<br>80.31	80.06<br>80.34	80.07<br>80.37	80.07<br>80.40	80.08<br>80.42
29	*30*	*31*				
80.09<br>80.45	Unavailability of the actual data at the time of data collection					
